# A Winter Cold

**Published:** 1882-11

**Authors:** F. H. Bosworth

**Affiliations:** Professor of Diseases of the Throat, in the Bellevue Hospital Medical College, New York


*FOR CONTENTS OF THIS NUMBER SEE PAGE* 624.

*THE*

### Independent Practitioner.

VOL. III.——*November*, 1882.——NO. 11.

### ORIGINAL COMMUNICATIONS.

*We are not responsible for the opinions expressed by contributors.*

ARTICLE I.

### A WINTER COLD.

BY F. H. BOSWORTH, M. D., N. Y. CITY.

Professor of Diseases of the Throat, in the Bellevue Hospital Medical College, New York.

(CONTINUED FROM PAGE 566.)

Exposure to cold then is the direct cause of the affection, but a prominent and indirect cause I believe to be in the very large majority of cases a chronic naso-pharyngeal catarrh. This may be of so mild a character that it gives rise to but moderate symptoms, yet it still exists as a powerful predisposing cause of a winter cold affecting the upper respiratory tract. So direct do I believe this connection to be between the chronic and acute affection that in many cases I am disposed to record as among the prominent symptoms of the chronic affection, constantly recurring acute attacks, the acute disease being really a symptom of the chronic. This fact I think is to be remembered, and becomes of importance in connection with the treatment.

In the management of a winter cold our first direction should be to enjoin absolute confinement to the house. This I regard of much importance as securing to the delicate and inflamed mucous tissues an equable temperature, and thus immunity from any causes which may act to aggravate the difficulty or retard the favorable resolution of the inflammatory process.

Remembering the causes as laid down above, which operate in the production of a cold, the first indication for treatment of course will be to supply as promptly as possible the deficiency caused by this loss of bodily heat. If this can be done in the early stages, when the secondary inflammatory process has not progressed, or better still, before it has set in, viz., during the preliminary febrile stage, the further progress of the disorder may be promptly arrested ; this constitutes what we generally call the abortive plan of treatment. This plan consists in short of producing copious perspiration. This perspiration be it remembered, however, is not primarily thc object it is desired to attain, but is simply the evidence that that object has been attained. The condition to be corrected is loss of bodily heat. The measures resorted to for this are measures which have a tendency to increase bodily heat. The evidence that this has been accomplished, viz., the restoration of this heat or even more, that an excessive heat is produced, is manifested by the perspiration. If this so-called sweating can be brought on in the early stage it serves the purpose of arresting the future progress of the trouble and putting an end to the inflammatory process. If it can be brought abour early in the progress of the inflammatory stage, its gravity can be very materially lessened, hence of course the earlier this abortive treatment is resorted to the better the result. The means of accomplishing this is by simple remedies familiar to all. A decoction of hot tea taken at bed time, with the addition of a foot bath and a moderate dose of Dover's powder, is all that is necessary, after which the body should be warmly covered in bed and extreme care exercised to prevent any exposure while the perspiration is going on. If the constitutional symptoms assume a graver form, that is, if the fever seems excessive, and the effect on the general system marked, much benefit in addition will be gained by the administration of ten grains of quinine in connection with the diaphoresis. It is generally asserted that following a copious perspiration there is danger of contracting additional cold on leaving the bed in the morning. This probably is a mistake, although the simple precaution should always be taken of allowing the body to cool off gradually before rising by removing a portion at a time of the bed covering, and also remaining in doors for a few hours after dressing. If, as the result of this treatment, all symptoms disappear, of course little else is needed except the exercise of ordinary precaution.

If, however, in spite of our abortive efforts the morbid process does not yield, our efforts must be directed towards controlling and directing it.

In order that our efforts in this direction shall be intelligently made, it is necessary that we understand the normal evolution of the inflammatory process. The first phenomenon that takes place when a mucous membrane becomes the seat of inflammation is an active congestion ; the vessels become enlarged and tortuous, the current of blood is retarded, and a condition of stasis occurs. In connection with this, the usual proliferations of cells is arrested, and the secretion of mucus ceases. The membrane becomes therefore abnormally dry. This lasts a few hours, or even days, when a new series of events sets in ; leucocytes breaks through the vessel coats, and serum escapes from the vessels into the surrounding tissues. At the same time an intense activity, which is merely an exaggeration of the normal physiological processes, pervade the inflamed part, apparently set in action by the escape of the leucocytes. New cells are developed, the glands secrete increased quantities of mucus, which with the serum escaping from the blood vessels, goes to make up the large amount of discharge which is poured out upon the surface of the membrane. The mucus membrane then in this stage is moist and coated with an abundant mucus of a grayish color, and semi-translucent.

In the further progress of the affection, the cell activity in the membrane continuing, we have a copious admixture of young, unripe or pus cells in the discharge, and there sets in a yellow, opaque muco-purulent discharge.

In the normal evolution of a winter cold, these conditions follow each other in regular order, but in many cases, as the result of a fresh cold, or some indiscretion, we find this regularity interfered with, and the membrane suddenly becomes congested and dry after the stage of discharge has set in. In other words, after a cough has "loosened," as it is called, it "tightens" up again.

For our present purpose it is sufficient merely to recognize the dry stage and the moist or secreting stage of a cold, and regulate our measures of relief accordingly. These measures consists in local applications and internal medication. In the dry stage we desire to stimulate the membrane to freer secretion. For this purpose we administer expectorants. We possess two remedies which, above all others, have an especial action on the upper air passage, and whose action, I think, is purely that of an expectorant; these are cubebs and ammonia. Just how these remedies act I do not propose to discuss at length. They are probably absorbed into the blood and eliminated through the mucous membrane of the air tract, thus serving to stimulate the membrane to a freer secretion of mucus. In whatever form the ammonia is administered, it is probably changed into the carbonate. In the cubeb berry there is present a volatile acid, the cubebic acid, whose action is much like that of ammonia. In administering an expectorant, I think the time-honored custom of giving a syrup is objectionable. The syrup is liable to be stale, it is usually nauseating to the taste, and moreover is liable to disorder the stomach. This is to be avoided especially, as the stomach and upper air passages are in close sympathy, and a disordered stomach is quite liable to have a deleterious effect on these parts. In glycerine we possess not only an admirable vehicle for the administration of remedies, but also an agent which exerts an exceedingly favorable influence on an inflammatory condition of the air passages. It is well in probably most, if not all cases, then, to substitute the glycerine for the syrup in our cough mixtures. In addition to the above it is desirable to add some mild anodyne to control the cough, allay irritation, while at the same time it does not check secretion. For this purpose I would prefer the dilute hydrocyanic acid. This is, I think, perhaps better than the Prunus Virginianus, whose main virtue, I take it, lies in the small amount of prussic acid that it contains. Bromide of potassium, or sodium, is another remedy which possesses a mild stimulating action on the air passages, in addition to its sedative action on the nerve centers. Basing our administration of remedies in the earlier and dry stages of a cold on this ground, we may give the following:

℞.

Acidi hydrocyanic. dil., ♏ xxiv.
Tinct. cubeba, ℨ ij.
Glycerinæ, ad ℥ ij.
M. Sig. One teaspoonful every 3 hours.

Or,

℞.

Potass. Bromidi, ʒ ij.

Ammoniæ Muriat., ʒ iiss.

Glycerinæ, ad. ℥ ij.

M. Sig. One teaspoonful every 3 hours.

After the dry stage of a cold has passed, and the discharge becomes free, in other words, when the cough has loosened, I believe we get the best results by the administration of those medicines which check secretion and allay cough and irritation. For this purpose I believe we possess no drug whose action combines these qualities to the same extent as opium, given in small doses. The vehicle for its administration being still by preference glycerine. A favorite prescription is as follows :

℞

Chlorodyne, ʒ i.
Glycerinæ, ad. ℥ ij
M. Sig. One teaspoonful every 3 hours.

There are, of course, other remedies which may be used, either separately or in combination with the above, such as hyoscyamus, chloral,etc.,with the preparations of tolu, sanguinariæ, glycyrhize, etc., but their action is somewhat vague, and it seems to me uncertain.

In addition to the internal use of drugs I think very much can be accomplished by local applications.

In the early dry stage much benefit will be gained by vapor inhalations. I know of none better than the following :

℞

Ol. Pics, ʒ i.

Tinct. Benzoini Co., ℥ ij

Potass Sulph. ʒ ss

M.

One teaspoonful of this is to be placed in a tea cup, and the cup filled with boiling water. This is to be held before the mouth and the vapor inhaled deeply and expired through the nose. This is to be repeated night and morning.

Among other drugs of a volatile nature which may be useful in this stage are: Turpentine, tinct. iodini, ol. cubeba and kreasote, to be used in the same way, about one teaspoonful in a half pint of hot water.

As the cough loosens the hot inhalations should be abandoned, and spray inhalations used. An excellent device for the purpose is the Codman and Shurtleff steam atomizer. This instrument I consider mischievous in chronic catarrhal affection, but in acute affection is of undoubted value.

With the freer secretion our efforts should be to control and arrest it. While the discharge is of a mucus character it is simply an evidence that the blood vessels are relieving themselves, and that the infiltrated tissues are pouring out their mucus and young cells. We desire here to control this process, and prevent, if possible, the setting in of the muco-purulent discharge of the last stage. We should, therefore, use the mild slightly astringent alkalies. The fluid being rendered alkaline in order that it may serve to more thoroughly cleanse the membrane and dissolve the mucus coating it. For this purpose we may use one of the following:

℞

Acidi Salicylic, gr. iv.
Sodæ Bicarb., gr. xij.
Aquæ, ℥ iv.
M.

℞

Sodæ Benzoat, ʒ i.

Sodæ Bicarb., ʒ i.

Aquæ, ℥ vi.

M.

℞

Acidi Boracic, ʒ i.

Sodæ Bicarb., ʒ ss.

Aquæ, ℥ vi.

M.

To be used by inhalation, night and morning, with the steam atomizer. Each sitting being limited to two to three minutes.

As there developes the stage of profuse muco-purulent discharge there should be used in the same manner the more decided astringents. As follows :

℞

Acidi tannic, Gr. x.
Aquæ, ℥ i.
M.

Or—

℞

Potass. Chlorat., gr. x.
Aquæ, ℥ i.
M.

Or—

℞

Alumini, gr. v.
Aquæ, ℥ i.
M.

Or indeed any of the simple unirritating astringents.

If our efforts still fail of controlling the progress of the disorder, we will find the muco-purulent character of the discharge changing to a greyish opaque discharge in which the pus cell is less frequent. The cough continues, and the affection is in danger of lapsing into a more or less chronic cough. In this stage I regard the value of local agents as very great. I would advise that now cold inhalations be used, and the steam atomizer be laid aside. Cold inhalation may be given through the large globe inhaler, but the atomization should be accomplished by the compressed air apparatus or the air bulbs.

We should now use daily one of the following, giving decided preference to the nitrate of silver, which in weak solution I regard as among the very best astringents we possess :

℞

Argenti nitrat, gr. ii.
Aquæ, ℥ vi.
M.

Or—

℞

Zinci Chloridi, gr. ii.
Aquæ, ℥ vi.

In connection with this I think it is of especial importance that the naso-pharyngeal and laryngeal cavities be treated, and on much the same principles as those above outlined. The application should be made directly to the parts, of course by atomization. These parts are within direct reach of our local remedies, while the trachea and bronchi can only be reached by inhalation.

It may be noticed that nothing is said about constitutional and general remedies. I have nothing to advance on this part of the subject that can be of especial value. Iron, quinine and general tonics will be given according as the indications are prominent in the view of the individual physician in charge of the case. I will only say that I regard the use of Tinct. ferri chloridi of especial value in these cases of winter cold, and almost invariably prescribe it as follows:

℞

Tinct. ferri chloridi, ʒ ii.
Glycerinæ, ad ℥ ij.
M. Sig. One teaspoonful before each meal.

The administration of laxatives and purgatives in winter cold also may be left to the judgment of the physician as of other general medication. The object of the paper has been to bring forward the importance of recognizing the stages of evolution of an inflammatory process in a mucous membrane and adapting our remedies thereto.

To summarize our whole subject:

1. A cold is usually ushered in by chilly sensations with fever, and if possible we should abort it at this stage by quinine, hot drinks and a sweat.

2. Failing this, the first stage of the inflammatory process which results from the cold is one of congestion and dryness. This cannot be arrested as a rule, and we therefore give expectorants internally, and make mildly stimulant local applications by inhalation and otherwise in order to promote the progressive evolution of symptoms.

3. The membrane having been relieved by the secreting stage setting in, we now control the process by using mild anodynes internally and making applications of local astringents.

4. If a cold relapses, or as it is said, "tightens up" again, we revert to our expectorants and local stimulants.

